# Betulinic Acid Derivatives NVX-207 and B10 for Treatment of Glioblastoma—An *in Vitro* Study of Cytotoxicity and Radiosensitization

**DOI:** 10.3390/ijms151119777

**Published:** 2014-10-30

**Authors:** Matthias Bache, Stephan Bernhardt, Sarina Passin, Henri Wichmann, Anja Hein, Martin Zschornak, Matthias Kappler, Helge Taubert, Reinhard Paschke, Dirk Vordermark

**Affiliations:** 1 Department of Radiotherapy, Martin Luther University Halle–Wittenberg, Ernst Grube Straße 40, D-06120 Halle, Germany; E-Mails: s.bernhardt@dkfz-heidelberg.de (S.B.); Sarina.Passin@web.de (S.P.); wichmann.henri@medizin.uni-halle.de (H.W.); anjahn@hein-reica.de (A.H.); mc_scop@gmx.net (M.Z.); dirk.vordermark@medizin.uni-halle.de (D.V.); 2 Division of Molecular Genome Analysis, German Cancer Research Center (DKFZ), Im Neuenheimer Feld 580, D-69120 Heidelberg, Germany; 3 Department of Oral and Maxillofacial Plastic Surgery, Martin Luther University Halle–Wittenberg, Ernst Grube Straße 40, D-06120 Halle, Germany; E-Mail: matthias.kappler@medizin.uni-halle.de; 4 Clinic of Urology, Friedrich Alexander University Hospital Erlangen, Hartmann Str. 14, D-91054 Erlangen, Germany; E-Mail: Helge.Taubert@uk-erlangen.de; 5 Biozentrum, Martin Luther Universität Halle–Wittenberg, Weinbergweg 22, D-06120 Halle, Germany; E-Mail: reinhard.paschke@biozentrum.uni-halle.de

**Keywords:** betulinic acid derivatives, glioma, cytotoxicity, irradiation, normoxia, hypoxia

## Abstract

Betulinic acid (BA), a pentacyclic triterpene, represents a new therapeutic substance that has potential benefits for treating glioblastoma. Recently, new strategies for producing BA derivatives with improved properties have evolved. However, few studies have examined the combination of BA or BA derivatives using radiotherapy. The effects of two BA derivatives, NVX-207 and B10, on cellular and radiobiological behavior were analyzed using glioblastoma cell lines (U251MG, U343MG and LN229). Based on IC_50_ values under normoxic conditions, we detected a 1.3–2.9-fold higher cytotoxicity of the BA derivatives B10 and NVX-207, respectively, compared to BA. Incubation using both BA derivatives led to decreased cell migration, cleavage of PARP and decreased protein expression levels of Survivin. Weak radiation sensitivity enhancement was observed in U251MG cells after treatment with both BA derivatives. The enhancement factors at an irradiation dose of 6 Gy after treatment with 5 µM NVX-207 and 5 µM B10 were 1.32 (*p* = 0.029) and 1.55 (*p* = 0.002), respectively. In contrast to BA, neither NVX-207 nor B10 had additional effects under hypoxic conditions. Our results suggest that the BA derivatives NVX-207 and B10 improve the effects of radiotherapy on human malignant glioma cells, particularly under normoxic conditions.

## 1. Introduction

Glioblastoma is the most common malignant primary brain tumor. Its treatment typically consists of surgery and subsequent radiotherapy with concomitant and sequential chemotherapy. Because glioblastomas are usually only incompletely resected, a definitive cure is not possible. In randomized trials, the additional use of temozolomide, a DNA alkylating agent, has improved the survival of patients with glioblastoma. Despite multimodal treatment concepts, the median overall survival has been estimated to be approximately 12 to 15 months [1,2,3,4]. The intrinsic resistance to DNA alkylating agents, such as temozolomide, represents another problem. Betulinic acid (BA), a pentacyclic triterpene, is a natural substance derived from birch bark and is used in various preparations as a medication for treating inflammation, malaria and obesity. Additionally, BA seems to be important for tumor therapy. *In vitro* analyses have indicated that BA is cytotoxic in various types of tumors, such as neuroectodermal tumors, head and neck cancer, colon cancer, lung cancer, ovarian cancer, melanoma and sarcoma [5,6].

BA is an effective agent for treating primary and established glioblastoma cell lines [7,8,9]. Initial investigations have revealed that BA improved the effects of chemotherapy and radiotherapy and increased doxorubicin- or cisplatin-induced apoptosis in various tumor cell lines [10]. The chemotherapy-resistant colon carcinoma cell line SNU-C5 showed significantly increased cytotoxicity when 5-fluorouracil, irinotecan or oxaliplatin treatment was combined with BA [11]. Using a combination of BA and irradiation, first-line trials with certain melanoma and head/neck-tumor cell lines illustrated additive effects [12,13]; this finding was confirmed by our results. In human malignant glioma cells, the additive effects under normoxic conditions were observed. Cytotoxicity and radiosensitization of BA were increased under hypoxic conditions [14].

**Figure 1:**
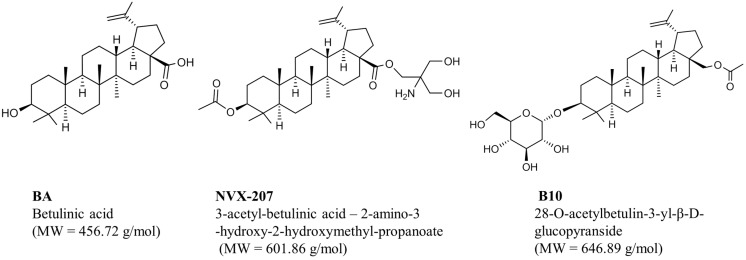
Structures of betulinic acid (BA), NVX-207 and B10.

A drawback for the therapeutic use of BA may be its low solubility. To overcome this limitation, several studies have investigated new derivatives evolved by modifying BA at the C-3 and C-28 positions [15,16,17]. Clinical testing is most advanced for bevirimat, a BA-derivative that is used against HIV. A clinical phase II study showed that the oral uptake was well tolerated by patients and that the viral load was effectively reduced compared with other antiviral substances [18]. NVX-207, a BA ester derivative, has a higher cytotoxicity than BA in various human and canine tumor cell lines. Furthermore, in a phase I/II study, the clinical use of NVX-207 led to complete remission of therapy-resistant tumors in dogs [19]. Another analysis indicated that glycosylation of BA significantly increased the activity and selectivity toward cancer cell lines [20]. B10, a BA ester derivative coupled with D-glucose, was more effective than BA and induced apoptosis in different tumor cell lines [21]. Recently, it was reported that B10 induced autophagy, in addition to cell death, by apoptosis in glioma cell lines [22]. However, to date, only a few studies have examined the combination of BA or BA derivatives with radiotherapy. In the present study, we analyzed the effects of the BA derivatives NVX-207 and B10 on cytotoxicity, migration, protein expression of PARP, Survivin and CAIX and radiosensitivity under normoxic and hypoxic conditions in human malignant glioma cells. The chemical structures of BA derivatives NVX-207 and B10, shown in Figure 1, were previously described [21,23].

## 2. Results and Discussion

### 2.1. Results

#### 2.1.1. Effects of Betulinic Acid (BA) and BA Derivatives on the Cytotoxicity and Protein Expression of Malignant Glioma Cell Lines under Normoxic and Hypoxic Conditions

**Figure 2:**
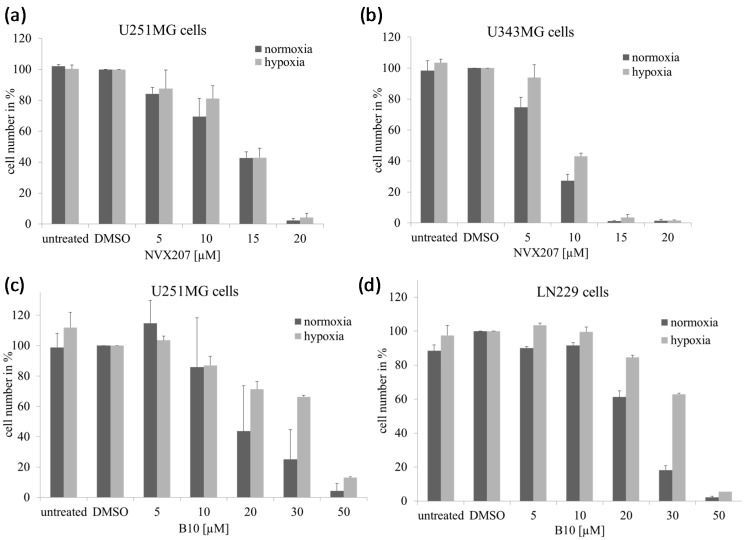
Effects of NVX-207 and B10 on the cell growth of glioma cells. Cell number of glioma cells after treatment with NVX-207 or B10 for 24 h under normoxic or hypoxic conditions. Under hypoxia, NVX-207 (**a**,**b**) and B10 (**c**,**d**) did not show decreased cell growth in glioma cell lines compared to normoxic conditions. The data represent the mean values (±SD) of three independent experiments.

**Figure 3:**
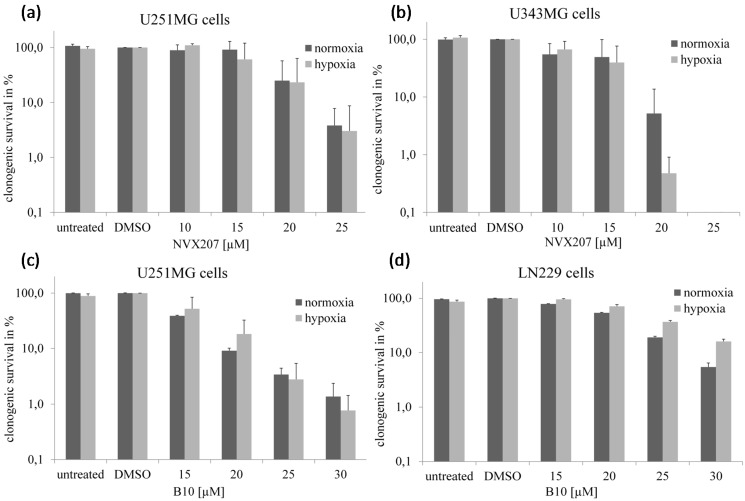
Effects of NVX-207 and B10 on the clonogenic survival of glioma cells. Clonogenic survival of glioma cells after treatment with NVX-207 or B10 for 24 h under normoxic or hypoxic conditions. Under hypoxia, NVX-207 (**a**,**b**) and B10 (**c**,**d**) did not show decreased clonogenic survival in glioma cell lines compared to normoxic conditions. The data represent the mean values (±SD) of three independent experiments.

BA showed a moderate cytotoxicity, with IC_50_ values ranging between 18.4 and 23.1 µM (Table 1). NVX-207 and B10 had a higher cytotoxicity than BA, with IC_50_ values ranging from 7.6–8.5 and 8.1–17.2 µM, respectively, in the three analyzed malignant glioma cell lines under normoxic conditions using the Sulforhodamine-B (SRB) assay. Under hypoxic conditions, a significantly increased cytotoxicity of BA, with IC_50_ values ranging from 7.0–8.5 µM, was observed (Table 1). Similar effects were previously observed using a clonogenic survival assay [14]. However, neither NVX-207 nor B10 had obviously stronger effects under hypoxic conditions. Overall, NVX-207 revealed the strongest effect, with IC_50_ values ranging from 7.6–9.6 µM in malignant glioma cells independent of the oxygen concentration. In U251MG cells, B10 presented a good response, with an IC_50_ value of 11.4 µM under hypoxic conditions. However, B10 displayed only moderate effects, with IC_50_ values of 22.4 and 24.9 µM in cell lines U343MG and LN229, respectively, under hypoxic conditions. Similar effects were observed using cell growth and clonogenic survival assays (Figure 2 and Figure 3).

**Table 1:** IC_50_ values of BA, NVX-207 and B10 for glioma cell lines, determined using the SRB-assay.

Cell Lines	IC_50_ (µM)					
	U251MG		LN229		U343MG	
	N	H	N	H	N	H
BA	18.4 ± 3.7	7.6 ± 1.0	23.1 ± 5.3	8.5 ± 0.7	20.2 ± 4.4	7.0 ± 1.2
NVX-207	7.6 ± 0.4	9.6 ± 0.9	7.9 ± 0.5	8.5 ± 0.3	8.5 ± 0.8	8.3 ± 0.1
B10	8.1 ± 0.8	11.4 ± 3.7	17.2 ± 1.5	22.4 ± 3.7	15.8 ± 1.7	24.9 ± 4.8

N, normoxia; H, hypoxia; IC_50_, inactivation concentration of 50%.

**Figure 4:**
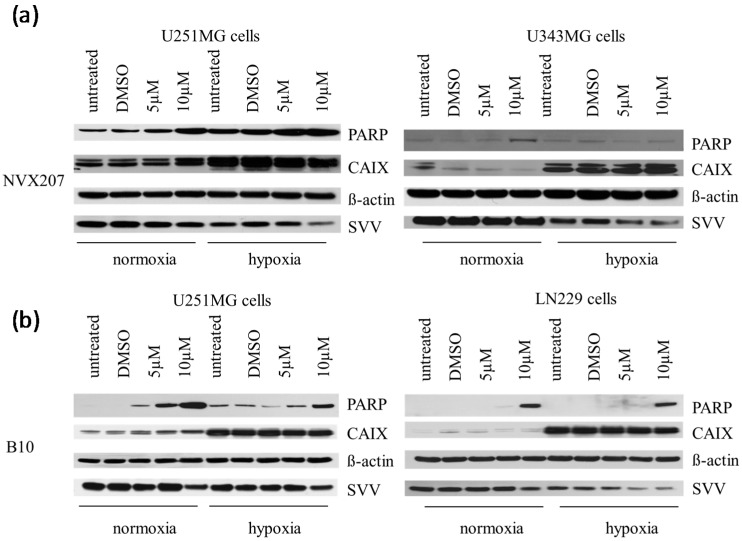
Effects of NVX-207 and B10 on protein expressions of PARP, CAIX and Survivin in glioma cells using Western blot analysis. NVX-207 and B10 treatment affects cleavage of PARP and expression of Survivin in glioma cells. However, there was no change in the expression levels of hypoxia-induced CAIX. β-Actin served as an internal loading control. Cell lines were untreated or treated using DMSO or increasing doses of NVX-207 (**a**) or B10 (**b**) under normoxic or hypoxic conditions. The Western blot analysis showed one representative result of three independent experiments.

Previously, we detected BA incubation results in the cleavage of the apoptotic protein PARP and a decrease in the protein level of the apoptosis inhibitor Survivin [14]. Using Western blot analysis, we examined the effects of BA derivatives on the cleavage of PARP as well as the expression levels of Survivin and hypoxia-induced CAIX under normoxic and hypoxic conditions (Figure 4). Hypoxia resulted in an increased CAIX expression level. Additionally, slightly increased levels of cleaved PARP or decreased expression levels of Survivin were detected under hypoxic conditions (Figure 4). Incubation with NVX-207 and B10 led to PARP cleavage and to a decrease in Survivin expression levels under normoxic and hypoxic conditions. However, no change in the hypoxia-induced CAIX protein level was observed (Figure 4). There were no effects of NVX-207 and B10 incubation on the mRNA expression levels of HIF1α and CAIX (data not shown). Our former analysis indicated that the stronger cytotoxic effects by BA under hypoxic conditions were accompanied by reduced hypoxia-induced gene expressions in U251MG and U343MG cells [14].

#### 2.1.2. Effects of BA and BA Derivatives on Necrosis, Migration, and Radiosensitivity in Malignant Glioma Cell Lines

**Figure 5:**
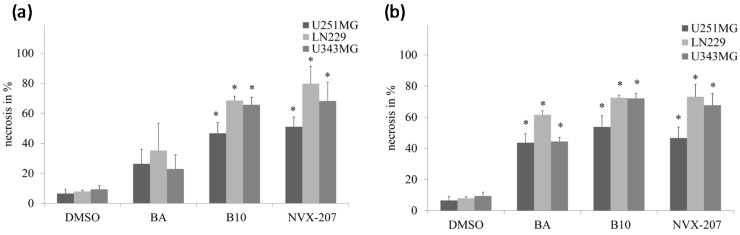
Effects of 20 (**a**) and 30 µM (**b**) BA, B10 or NVX-207 on necrosis of glioma cell lines were determined using the LDH assay. The data represent the mean values (±SD) of three independent experiments. Significant differences between BA and BA derivatives were labeled (* *p* < 0.05).

NVX-207 and B10 induce higher rates of necrosis compared to BA, as detected by the LDH assay. A significantly higher rate of necrosis of glioma cell lines was detected using 20 µM NVX-207 and B10. BA also showed a significant increase of necrosis at a concentration of 30 µM (Figure 5).

**Figure 6:**
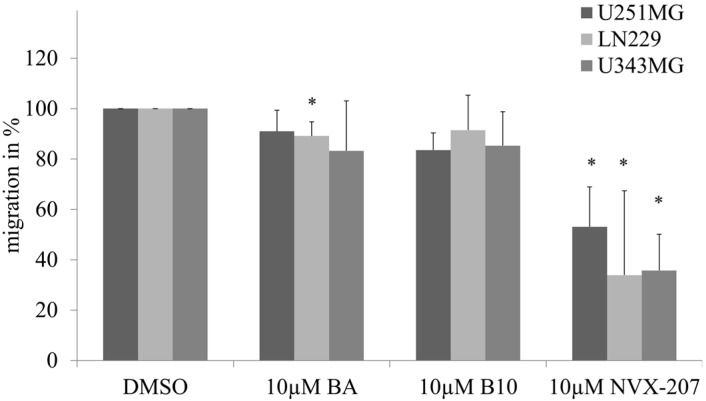
Effects of 10 µM BA, NVX-207 or B10 on the migration of glioma cells. The data represent the mean values (±SD) of three or four independent experiments (* *p* < 0.05).

The effects of NVX-207, B10 and BA on the cell migration rates of malignant glioma cell lines were determined using a scratch assay. A dose-dependent inhibition of migration was observed. At a concentration of 5 µM NVX-207, B10 or BA, a slightly decreased rate of migration between 8% and 23% was detected (data not shown). In all of the glioma cell lines, NVX-207 showed the strongest effects on the inhibition of migration at a concentration of 10 µM compared to B10 and BA (Figure 6).

**Figure 7:**
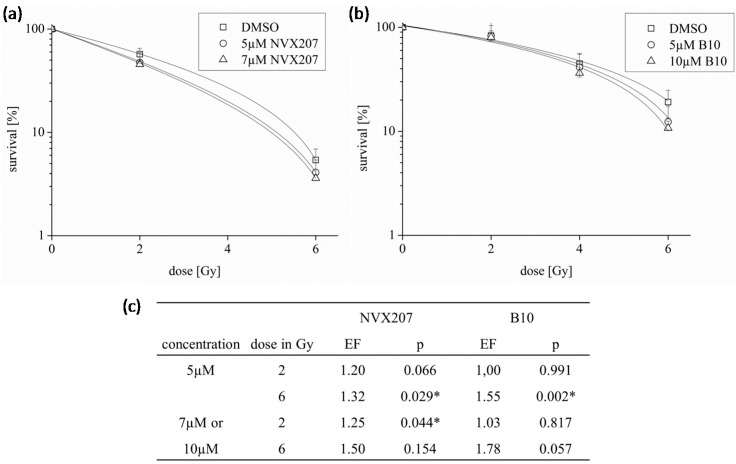
Effects of NVX-207 and B10 on the radiosensitivity of glioma cells. U251MG cells were treated with NVX-207 (**a**) or B10 (**b**) and irradiated with a dose of 2 and 6 Gy under normoxic conditions (**c**). The data represent the mean values (±SD) of three independent experiments (* *p* < 0.05). EF, enhancement factor.

We determined the radiosensitivity of NVX-207 and B10 after irradiation with 2 or 6 Gy. The two BA derivatives had slightly increased effects on the radiosensitivity of glioma cells. In U251MG cells, the enhancement factors for 5 µM NVX-207 or B10 after a radiation dose of 6 Gy were 1.32 (*p* = 0.029) and 1.55 (*p* = 0.002), respectively. The enhancement factors increased with higher concentrations of both BA derivatives (Figure 7). Former analyses have shown that BA slightly radiosensitized glioma cells [14].

### 2.2. Discussion

Betulinic acid represents a new therapeutic substance that has potential benefits for treating glioblastomas. However, low solubility has been observed to prevent the therapeutic application of BA for tumor therapy. Modifications could be an alternative approach for using BA in therapeutic applications. In the present study, we analyzed the cell biological effects of two promising BA derivatives in combination with radiation therapy on human malignant glioma cell lines.

Compared with BA, the two BA derivatives, NVX-207 and B10, showed a 1.3–2.9-fold higher cytotoxicity in glioma cell lines under normoxic conditions (Table 1). Additionally, NVX-207, a betulinic ester, displayed the strongest effect independent of the oxygen concentration. Recently, NVX-207 revealed strong cytotoxicity in tumor cells *in vitro* and *in vivo* [19]. The gene expression analyses have suggested that NVX-207 has modulatory effects in lipid metabolism, with an induction of insulin-induced gene 1, which is a key regulator of sterol synthesis [19]. Nevertheless, additional information concerning the molecular mechanisms and targets is needed to specifically design new anticancer drugs, including BA derivatives [24]. In a former study, the 3-*O*-glycoside B10 showed higher cytotoxicity in different tumor cell lines compared to BA [21]. We confirmed these findings for malignant glioma cell lines under normoxic conditions in our study (Table 1). The high activity of B10 is triggered by apoptotic and non-apoptotic cell death in glioma cell lines [22]. Consistent with this finding, our analysis showed that B10 induced the cleavage of the apoptotic protein PARP and decreased the protein level of the apoptosis inhibitor Survivin. By analyzing the release of LDH, we observed a higher rate of necrosis after NVX-207 and B10 incubation compared to BA (Figure 5). Additional analysis revealed that the effectiveness of the anticancer activities of 3-*O*-glycosides was varied based on the sugar at the C-3 position and the modification of the C-28 position [20,25].

Comparable to BA, NVX-207 and B10 treatments resulted in slightly reduced rates of glioblastoma cell migration. However, NVX-207 showed the strongest effect on inhibition of migration (Figure 6). To date, few studies have examined the combination of BA or BA derivatives with radiotherapy. NVX-207 and B10, in combination with irradiation, have shown slightly enhanced effects on the radiosensitivity of malignant glioma cells (Figure 7). Our results have shown that these two treatments were more effective in combination. Consistent with this finding, the preliminary data on incubation with B10 showed additive effects with the PI3K inhibitor GDC-0941, temozolomide or irradiation [26,27]. Considering BA in combination with radiation, few studies obtained additive or radiosensitizing effects in different tumor cell lines [12,13,14]. These results suggest that BA and BA derivatives are potentially useful in combination with radiotherapy. However, this observation must be examined more closely in future studies. Furthermore, the incubation of BA resulted in HIF1α inhibition or anti-angiogenic activity [28,29,30]. Consistent with this finding, we previously demonstrated a reduced, BA-induced HIF1α expression level that was coupled with stronger effects on cytotoxicity and radiosensitivity under hypoxic conditions in glioma cells [14]. However, NVX-207 and B10 did not affect hypoxia-induced gene expression of HIF1α or CAIX or cytotoxicity dependent on the oxygen concentration. The results of SRB assay and cell growth analysis suggest that B10 decreases cytotoxicity under hypoxic conditions. It is well known that cell growth is strongly delayed under hypoxic conditions. Lesser proliferation of hypoxic control cells seems to be responsible for the apparent resistance of glioma cell lines. Contrary to this observation, B10 had a stronger effect on cytotoxicity in two glioma cell lines under hypoxic conditions [27]. Potentially, the cell-type specificity or the different *p53* gene status of the analyzed cell lines [31,32] could explain the different results.

## 3. Experimental Section

### 3.1. Cell Lines, Culture Conditions, and Treatments with BA and Irradiation

The human malignant glioma cell lines U251MG (kindly provided by Dr. Ariane Söling, Department of Pediatrics, University Göttingen, Göttingen, Germany), U343MG (CLS Cell Lines Service, Eppelheim, Germany) and LN229 (kindly provided by Annie-Claire Diserens, Laboratoire de Neurochirurgie, Lausanne, Switzerland) were grown in RPMI 1640 medium (Lonza, Walkersville, MD, USA) containing 10% fetal bovine serum (PAA Laboratories, Cölbe, Germany), 1% sodium pyruvate (Invitrogen, Karlsruhe, Germany), 185 U/mL penicillin (Invitrogen) and 185 μg/mL streptomycin (Invitrogen) at 37 °C in a humidified atmosphere containing 3% CO_2_. Hypoxia (<0.1% O_2_) was achieved using a gas generator system as previously described [33]. All of the experiments were performed with cells in logarithmic growth phase. BA, NVX-207 and B10 (all compounds from BioSolution GmbH, Halle, Germany) were dissolved in dimethyl sulfoxide (DMSO) to achieve a 20 mM stock solution (Figure 1). The cells were seeded in 25-cm^2^ flasks 24 h before treatment; the cells were treated with BA, NVX-207 or B10 for 24 h at 37 °C under normoxic or hypoxic conditions and were subsequently irradiated in tissue culture flasks (Greiner, Frickenhausen, Germany). Irradiation was performed using 6-MV photons, adequate bolus material, and a linear accelerator (SIEMENS ONCOR, Erlangen, Germany) at a dose rate of 2 Gy/min.

### 3.2. Sulforhodamine-B (SRB) Assay

Cytotoxic activities were evaluated using the Sulforhodamine-B (SRB) assay. Exponentially growing cells were seeded into 96-well plates at cell densities to prevent confluence for 96 h. After 24 h, the cells were treated using a dilution series of the compounds for 72 h under normoxic or hypoxic conditions. After treatment, the adherent cells were fixed using 10% TCA at 4 °C for 1 h; the cells were washed with ice-cold water and were dyed using 100 µL of 4.4% SRB solution for 10 min. After staining, the plates were washed with 1% acetic acid and air-dried overnight. Three hundred microliters of 20 mM Tris base solution was added, and the absorbance was measured at 540 nm using a 96-well plate reader (TECAN GENios, Männedorf, Switzerland). The IC_50_ values indicate the concentrations of the compound that cause 50% cell inhibition. The data were obtained in three independent experiments.

### 3.3. Lactate Dehydrogenase (LDH) Assay

To detect cellular membrane damage with the release of the cytosolic enzyme LDH into the culture medium, a colorimetric LDH assay was performed. The cells were seeded for 24 h and were treated with dilutions of the compounds. After a 24-h incubation of the compounds, 50 μL of the culture supernatant was collected from each well and was placed in a 96-well plate. The LDH activity was assessed using a CytoTox-ONE™ Homogeneous Membrane Integrity Assay (Promega, Madison, WI, USA) according to the manufacturer’s instructions. Briefly, 50 µL of CytoTox-One solution was added to the samples, which were then incubated in the dark at room temperature for 15 min. After incubation, the reaction was terminated with 25 µL of stop solution, and the excitation at 560 nm/emission at 590 nm was measured using a microplate reader. As a positive control for LDH release, the cells were treated using 1% solution of Triton X-100 (Sigma–Aldrich, Munich, Germany) for 5 min to lyse all of the cells before collecting the supernatants. Sample emission values were normalized to maintain a positive control and culture medium background. All of the experimental conditions were performed in three independent experiments.

### 3.4. Scratch Assay

We used a wound-scratch assay to determine the migration of cells after treatment with BA, NVX-207 or B10. The cells were grown in 24-well cell culture plates in RPMI medium containing 10% FBS and cultured to 100% confluence. A uniform, cell-free area was created by scratching the confluent monolayer with a 200-µL pipette tip. To determine the migration of glioma cells, the wound closure was observed 16 h after incubation with BA, NVX-207 or B10. To evaluate the relative cell migration rate, the initial and the final wound areas were measured using Adobe Photoshop CS6. The wound-scratch assay was performed three or four times in independent experiments.

### 3.5. Western Blotting

The cells were washed, trypsinized and centrifuged. The cells were washed with PBS and resuspended in 100 µL of lysis buffer (50 mM Tris at pH 8.0, 0.3 M NaCl, 1 mM EDTA, 0.5 mM dithiothreitol, 0.1% NP40 and protease inhibitors) followed by ultrasonic homogenization. After centrifugation at 14,000× *g* for 15 min, the supernatant was collected, and the protein concentration was determined using the Bradford assay (BioRad, Munich, Germany). Approximately, 30 mg of total protein from each cell lysate was separated on a 10% NuPAGE Bis–Tris (Invitrogen) gel that was placed in an X-Cell SureLock Mini-Cell (Invitrogen). The membrane was blocked with 10% non-fat milk in TBST (50 mM NaCl, 30 mM Tris–HCl at pH 8.0 and 0.1% Tween) for 1 h and incubated with rabbit anti-human Survivin antibody (1:1000 dilution, clone AF886, R&D Systems, Wiesbaden, Germany), rabbit anti-human cleaved PARP (1:2000, Cell Signaling, Danvers, MA, USA), mouse CAIX antibody (1:3000, BioScience, Bratislava, Slovakia) and mouse anti-β-actin (1:5000, Sigma, Deisenhofen, Germany) at 4 °C overnight. After washing, the membranes were incubated with a horseradish peroxidase-labeled goat anti-rabbit or anti-mouse IgG (1:2000, DAKO, Glostrup, Denmark) for 1 h at room temperature. For protein detection, the membranes were incubated with ECL substrate or the ECL Plus Blotting Detection System (Amersham Pharmacia Biotech, Freiburg, Germany) for 1 min and exposed to X-ray film (Biomax, Kodak, Braunschweig, Germany). To detect a difference between the expression levels of cleaved PARP, CAIX or Survivin we visually compared the differences between the DMSO-treated control cells and the NVX-207 or B10-treated cells. β-Actin served as an internal loading control. The Western blot analysis showed one representative result in three independent experiments.

### 3.6. Clonogenic Survival Assays and Radiosensitivity

The cells were trypsinized 24 h after treatment with BA, NVX-207 or B10 (1 h after irradiation), and the cell numbers were determined. Based on the optimal plating efficacy (depending on the BA treatment and irradiation dose), 500–10,000 cells were seeded in 25-cm^2^ flasks. The cells were cultured in RPMI supplemented with 10% FBS in a humidified atmosphere of 3% CO_2_ at 37 °C, and the medium was changed after 5 days. Between 10 and 14 days after irradiation, the cells were fixed using paraformaldehyde (Sigma, Deisenhofen, Germany), and colony formation was visualized by staining with 10% Giemsa solution (Sigma, Deisenhofen, Germany). Only colonies with >50 cells were scored to determine the surviving fraction (SF), which was defined as the ratio of colonies that were formed after irradiation with 2 or 6 Gy to the number of colonies that were formed in the unirradiated controls. The enhancement factor (EF) was defined as the ratio of the surviving fraction of the DMSO-treated control cells to B10- or NVX-207-treated cells and was dependent on the dose of irradiation. The data represented at least three independent experiments.

### 3.7. Statistical Analyses

The experimental results were analyzed using paired Student’s *t*-tests. A *p*-value of 0.05 was considered to be statistically significant.

## 4. Conclusions

In conclusion, the BA derivatives NVX-207 and B10 modulate clonogenic survival, migration, apoptosis and radiosensitivity in human malignant glioma cells. Compared to BA, higher cytotoxic effects were observed, particularly under normoxic conditions. These results suggest that BA derivatives may be capable of increasing the therapeutic efficacy of radiotherapy in malignant gliomas.
